# Acyclic Sesquiterpenes from the Fruit Pericarp of* Sapindus saponaria* Induce Ultrastructural Alterations and Cell Death in* Leishmania amazonensis*

**DOI:** 10.1155/2017/5620693

**Published:** 2017-08-22

**Authors:** Amanda Louzano Moreira, Débora Botura Scariot, Bruna Luíza Pelegrini, Greisiele Lorena Pessini, Tânia Ueda-Nakamura, Celso Vataru Nakamura, Izabel Cristina Piloto Ferreira

**Affiliations:** ^1^Programa de Pós-Graduação em Ciências Farmacêuticas, Universidade Estadual de Maringá, Avenida Colombo 5790, Jd. Universitário, Maringá, PR, Brazil; ^2^Laboratório de Inovação Tecnológica no Desenvolvimento de Fármacos e Cosméticos, Universidade Estadual de Maringá, Avenida Colombo 5790, Jd. Universitário, Maringá, PR, Brazil

## Abstract

Previous studies reported antiprotozoal activities of* Sapindus saponaria* L. The aim of this work was the evaluation of antileishmanial activity and mechanism of action of extract and fractions of* S. saponaria* L. Hydroethanolic extract (EHA) obtained from fruit pericarps was fractionated using solid-phase extraction in a reversed phase, resulting in fractions enriched with saponins (SAP fraction) and acyclic sesquiterpene oligoglycosides (OGSA fraction). The activities of EHA, SAP, and OGSA were evaluated by antiproliferative assays with promastigote and intracellular amastigote forms. Cytotoxicity on macrophages and hemolytic activity were also analyzed. Morphological and ultrastructural changes in* Leishmania amazonensis* promastigotes were evaluated by electron microscopy. Flow cytometry was used to investigate mitochondrial dysfunction and phosphatidylserine exposure. OGSA was more selective for parasites than mammalian J774A1 macrophage cells, with selectivity indices of 3.79 and 7.35, respectively. Our results showed that only the OGSA fraction did not present hemolytic activity at its IC_50_ for promastigote growth. Electron microscopy revealed changes in parasite flagellum, cell body shape, and organelle size, mainly mitochondria. Flow cytometry analysis indicated mitochondrial membrane and cell membrane dysfunction. OGSA showed antileishmanial activity, resulting in several changes to protozoa cells, including mitochondrial depolarization and early phosphatidylserine exposure, suggesting a possible apoptotic induction.

## 1. Introduction

*Sapindus saponaria* L. (Sapindaceae) is a tropical tree species that is regularly found within the north, northeast, and central west areas of Brazil. Its height ranges from 4 to 9 m, and it presents bright brown-colored fruits 2 cm in diameter [1].

*S. saponaria* L., easily accessible owing to its popularity, has diverse traditional uses in the treatment of diseases of the nervous, respiratory, and genitourinary systems, in addition to being used to heal skin illnesses and as a biocide. The fruits, bark, and root of this species are employed in folk medicine as an astringent, soothing, expectorant, diuretic, tonic, and antitussive agent [2]. Potential activities of* Sapindus* species, including antimicrobial, antidiabetic, molluscicidal, cytotoxic, fungicidal, and anti-inflammatory, are attributed to the presence of, among other substances, glycosides [3].

Fruits of* S. saponaria* L. possess various glycosides in their pericarps. There are 30 saponins derived from the hederagenin triterpene oleanolic acid and 63 acyclic sesquiterpene oligoglycosides, as shown by liquid chromatography with UV-detection and MS (LC/UV/ESI-MS) and MS/MS fragmentation studies [4].

Glycosides are also considered promising antiprotozoal agents, particularly against* Leishmania* spp. [5–9]. The protozoa that cause leishmaniasis are transmitted by sandflies (insects of* Lutzomyia* spp. and* Phlebotomus* spp.) that are intermediate hosts of flagellated promastigote forms of the parasite [10, 11].

An understudied disease, leishmaniasis, is prevalent in America, Africa, and Asia, mainly in developing countries, but also in Europe [12]. Despite its prevalence, few drugs have been applied to treat this illness, and high toxicity and pronounced side effects have been reported. Pentavalent antimonials, used as the first-line treatment for cutaneous leishmaniasis, are effective, but usually other limitations are observed, such the necessity for long-term treatment and the resulting difficulty in patient adherence [13–15].

Currently, elucidating the chemical composition of plant extracts, which are easily accessed by low-income populations, has become relevant to treating neglected diseases and discovering substances with new therapeutic mechanisms. Therefore, this study aimed to examine the antileishmanial activity and mechanism of action of extracts and fractions of* S. saponaria* L. on* L. amazonensis* parasites.

## 2. Materials and Methods

### 2.1. Obtaining the Hydroethanolic Extract, Acyclic Sesquiterpene Oligoglycosides, and Saponins of* S. saponaria* L. 

Fruits of* S. saponaria* L. were collected during maturation on campus at the State University of Maringá on September 14, 2012, and the vegetal material was identified by the Department of Botany, where the sample of this species was deposited as number HUM 11710. The fruits were washed and separated into pericarps and seed. Pericarps were subjected to maceration with occasional stirring until exhaustion, using an extraction solvent mixture of ethanol : water 9 : 1 (v/v). This solution was filtered, and the solvent evaporated under reduced pressure in a rotary evaporator at 40°C. The portion not volatilized in this process was lyophilized to become the hydroethanolic extract (EHA). To obtain two separate classes of glycosides, acyclic sesquiterpene oligoglycosides (called fraction OGSA) and saponins (called SAP fraction), 1000 mg of EHA was subjected to a solid-phase extraction in a cartridge with a reverse phase of octadecylsilane (ODS, Supelco Supelclean LC-18, 6 mL, 1000 g) under reduced pressure on a Supelco system. The eluents used were 5 mL aliquots of the mixtures of ACN (chromatographic grade) : H_2_O (purified with a Milli-Q system) in proportions (v/v) of 2 : 8, 3 : 7, 4 : 6, 5 : 5, 6 : 4, 7 : 3, and 10 : 0, in that order.

### 2.2. Glycoside Characterization by Mass Spectrometry with Electrospray Ionization (ESI-MS)

Analyses were performed on a mass spectrometer model MICRO MASS QUATTRO LC, equipped with triple quadrupole and electrospray ionization in negative mode. The samples were diluted in methanol (chromatographic grade) and inserted via direct injection (10 *μ*L), using nitrogen as the nebulization and desolvation gas. The capillary voltage values and cone extractor were optimized for each sample. The MS/MS experiments were performed using argon as collision gas: collision energy was 25–35 eV for saponins and 40–70 eV for OGSA. For equipment operation and collection and processing of data, Masslynx 3.3 software was used (Micro Mass).

### 2.3. Parasite and Macrophage Cultures

Promastigote forms of* Leishmania amazonensis* (MHOM/BR/75/Josefa) were cultivated at 25°C in Warren's medium, consisting of brain-heart infusion medium and 10 *μ*g/mL each of hemin and folic acid (pH 7.2), plus 10% inactivated fetal bovine serum (FBS; Gibco Invitrogen, New York, NY, USA). The macrophage lineage (J774A1) was maintained in RPMI 1640 medium (Gibco Invitrogen Co., Grand Island, NY, USA) with L-glutamine and 10% FBS, at 37°C in an atmosphere containing 5% CO_2_.

### 2.4. *In Vitro* Antiproliferative Assays

A suspension containing 1 × 10^6^ parasites/mL of promastigote forms in the logarithmic phase of growth was added in 24-well culture microplates with increasing concentrations of OGSA, SAP, and EHA (10, 50, 100, 250, and 500 *μ*g/mL) previously solubilized in dimethyl sulfoxide (DMSO) at a final concentration of 0.5% in all tests. For 72 h, these protozoa were maintained at 25°C in Warren's medium supplemented with 10% FBS. Antileishmanial activity was determined by directly counting free-living parasites in a Neubauer chamber to find the concentration that inhibited 50% (IC_50_) and 90% (IC_90_) of the parasite growth compared to untreated control cells.

Antileishmanial activity assays against intracellular amastigote forms were performed according to methods described by Kaplum et al. [16]. Briefly, peritoneal macrophages were isolated from BALB/c mice by washing the peritoneal cavity with cold phosphate-buffered saline (PBS) supplemented with 3% FBS (protocol number 029/2014 approved by the Ethical Committee of the State University of Maringa). A suspension of 5 × 10^5^ macrophages/mL was plated in a 24-well microplate and incubated for 2 h at 37°C in a 5% CO_2_ atmosphere. These cells were infected with promastigote forms in the stationary growth phase at a 7 : 1 parasite : macrophage ratio for 4 h at 34°C and 5% CO_2_. Afterwards, the infected macrophages were treated with OGSA, SAP, or EHA (1, 5, 10, 50, and 100 *μ*g/mL) and incubated for 48 h. The number of intracellular amastigotes was counted under a light microscope after Giemsa staining, in at least 200 macrophages for each condition.

### 2.5. *In Vitro* Cytotoxicity and Hemolysis Assays

J774A1 macrophage cells (5 × 10^5^ cells/mL) were added to each well in 96-well microplates and incubated in a 5% CO_2_ atmosphere at 37°C to obtain a macrophage monolayer. Increasing concentrations of OGSA (1, 5, 10, 50, 100, 250, or 500 *μ*g/mL) were added and incubated for 48 h in the same conditions. A colorimetric MTT method was applied to verify cell viability through the metabolization of MTT (3-[4,5-dimethylthiazol-2-yl]-2,5-diphenyltetrazolium bromide formazan; 2 mg/mL) to purple formazan crystals after 4 h of incubation in the dark. DMSO was used to solubilize formazan to facilitate analysis on a microplate reader (Bio-Tek Power Wave XS) at 570 nm [17]. The percentage of viable macrophages was calculated compared to the untreated cells and the concentration that resulted in 50% cell viability of the macrophages (CC_50_) was determined.

Human blood (type A+) was defibrinated using glass beads and a 6% erythrocyte suspension was prepared in PBS. Plant extracts and fractions were added to the erythrocyte suspension in a 96-well microplate (10, 50, 100, 500, or 1000 *μ*g/mL) and incubated for 3 h at 37°C. Next, the plates were centrifuged for 3 min at 1200 rpm, and 100 *μ*L of the supernatant was transferred to another sterile 96-well plate to read the absorbance at 550 nm in a microplate reader (Bio-Tek, model Power Wave XS). The nonionic surfactant Triton X-100 (1%), known to rupture lipid membranes, was used as positive control, and the negative control was a suspension of untreated red blood cells. This method measured hemoglobin release due to hemolysis.

### 2.6. Scanning Electron Microscopy

After treatment for 72 h at the IC_50_ or IC_90_ of OGSA, cells were fixed with 2.5% glutaraldehyde in 0.1 M cacodylate buffer for 3 h. Next, the cells were adhered to poly-L-Lysine coated plates, which were dehydrated in increasing percentages of ethanol from 30% to 100%. The samples were critical-point-dried in CO_2_ and then coated with gold alloy. The analysis was performed on a Shimadzu SS-550 scanning electron microscope to evaluate possible morphological alterations in the cell surfaces upon OGSA treatment [18].

### 2.7. Transmission Electron Microscopy

Promastigote samples were treated with the IC_50_ or IC_90_ of OGSA for 72 h at 25°C. Fixed samples were postfixed in a solution containing 1% osmium tetroxide, 5 mM calcium chloride, and 0.8% potassium ferrocyanide. At the end of this process, these parasites were dehydrated in graded acetone, embedded with epoxy resin, and then polymerized at 60°C. Ultrathin sections were obtained and stained with 5% uranyl acetate and lead citrate and observed on JEOL JEM 1400 transmission electron microscope to analyze ultrastructural cell aspects [18].

### 2.8. Determination of Parasite Cell Volume by Flow Cytometry

Promastigote forms were treated with OGSA (100, 450, or 900 *μ*g/mL) for 24 h at 25°C. These treated parasites were analyzed using a BD FACSCalibur flow cytometer and 10,000 events were interpreted by CellQuest Pro software, using forward scatter (FSC) as a parameter. Histograms were analyzed considering the median point. Actinomycin D (Sigma-Aldrich), a known antineoplasic drug that induces apoptosis, and consequently reduces cell volume, was used as a positive control [19, 20].

### 2.9. Determination of Mitochondrial Transmembrane Potential (ΔΨm) by Flow Cytometry

Promastigote forms were treated with OGSA at 100, 450, or 900 *μ*g/mL for 24 h at 25°C and incubated with 1 *μ*L (5 mg/mL in ethanol) rhodamine 123 (Rh 123; Sigma-Aldrich, St. Louis, MO, USA) for 15 min. Parasites treated with the protonophore carbonyl cyanide 3-chlorophenylhydrazone (CCCP; Sigma-Aldrich; 100.0 *μ*M) were used as a positive control. CCCP is a known mitochondrial uncoupler that allows proton transportation to the mitochondrial matrix, causing mitochondrial transmembrane potential depolarization [21]. Ten thousand events were analyzed using a BD FACSCalibur flow cytometer, and the data were analyzed on CellQuest Pro software [22]. Parasites exhibiting mitochondrial depolarization were represented in a histogram, using FL-2 as a parameter.

### 2.10. Detection of Phosphatidylserine Exposure by Flow Cytometry

Double staining for annexin-V-fluorescein isothiocyanate (FITC) and propidium iodide (PI) was performed using an annexin-V apoptosis detection kit according to the manufacturer's instructions. Briefly, untreated and OGSA-treated parasites (100, 450, or 900 *μ*g/mL for 24 h at 25°C) were resuspended in 500 *μ*L binding buffer (140 mM NaCl, 5 mM CaCl_2_, and 10 mM HEPES-Na, pH 4.7), and 5 *μ*L of FITC-conjugated Annexin-V was added. The labeled parasites were maintained at 25°C in the dark for 5 min. To complete the double staining, 400 *μ*L of binding buffer plus 50 *μ*L PI were added. Fluorescence of 10,000 events was analyzed on a BD FACSCalibur cytometer and results were interpreted by CellQuest Pro software, using FL-1 and FL-2 parameters. Miltefosine, an antineoplasic drug that induces autophagy, was used as a positive control [23]. Late apoptotic processes were represented by PI-positive parasites in upper quadrants and apoptotic processes by FITC fluorescence in upper and lower-right quadrants [24].

### 2.11. Statistical Analysis

In the cellular experiments, the results were expressed as the mean and standard deviation of at least three independent experiments. A Student's *t*-test was performed, and *p* values less than 0.05 were regarded as significant.

### 2.12. Ethics Statement

For the hemolytic assay, blood was obtained from healthy volunteer donors according to the Declaration of Helsinki (Ethical principles for medical research involving human subjects) last reviewed in 2008. The donors received an explanation about the purpose of the study and provided their written consent before blood collection. The blood was collected by brachial vein puncture by a trained professional with appropriate material and medical support. All procedures were conducted as described in specific protocol approved by the “Comitê de Ética em Pesquisa com Seres Humanos of the Universidade Estadual de Maringá” (acceptance 293/2006 COPEP-UEM). For the assays that involved mice macrophages, BALB/c mice were obtained from the Central Animal Facility of the Universidade Estadual de Maringá. All procedures were carried out in accordance with the guidelines established by the Committee on Ethics of Animal Experiments of the Universidade Estadual de Maringá, as stated in the detailed protocol approved for this experiment (acceptance 029/2014).

## 3. Results

### 3.1. Characterization of EHA, SAP, and OGSA Fractions

EHA, OGSA, and SAP solutions in methanol were inserted via direct injection into the mass spectrometer in negative mode, resulting in mass spectra similar to those obtained by Murgu & Rodrigues-Filho (2006). In the mass spectrum of EHA, peaks were observed from* m/z* of 400–1550. The region of acyclic sesquiterpene oligoglycosides was formed by intense peaks from* m/z* 1100–1550, and each peak was separated by 42 Da (equivalent to repeated losses COCH_2_). The region of saponins contained less intense peaks from* m/z* of 650–1000, and some peaks were also separated by 42 Da.

In the OGSA mass spectrum, there was a prevalent presence of peaks above* m/z* values of 1100, confirming the enrichment of the OGSA fraction with acyclic sesquiterpene oligoglycosides. The same result applied to the mass spectrum of the SAP fraction, in which the extensive presence of peaks below the* m/z* of 1100 was observed, confirming the enrichment of the SAP fraction with saponins.

A peak from each spectrum of the fractions (*m/z* 1187 of OGSA and* m/z* 923 of SAP) was selected for MS/MS experiments. Data provided by MS/MS confirmed the structure of the glycosides, as the sequence and molecular weight of sugars and number and types of acyl groups were linked to the substance. Data fragmentation of the ion* m/z* 1187 and* m/z* 923 agreed with the fragmentation pattern of the reference substances, according to data obtained by Murgu (2002) in* S. saponaria* L., Wong et al. (1991) in* Sapindus delavayi*, and Kasai et al. (1986) in* Sapindus mukorossi*.

### 3.2. Activity against* L. amazonensis* Forms, Cytotoxicity, and Hemolysis

**Figure 1:**
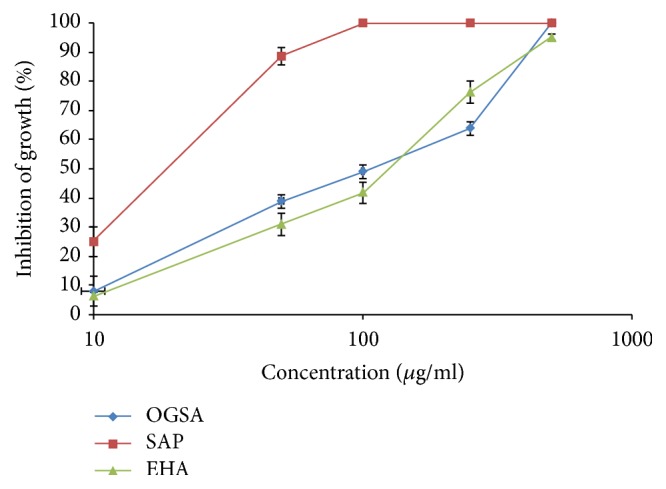
Effect of OGSA, SAP, and EHA on* L. amazonensis* promastigote forms after incubation of parasite for 72 h with 10, 50, 100, 250, and 500 *μ*g/mL.

EHA, OGSA, and SAP dose-dependently inhibited promastigote forms during 72 h of treatment, exhibiting IC_50_ values of 153.70 ± 3.20, 100.92 ± 1.56, and 25.41 ± 2.88 *μ*g/mL, and IC_90_ of 436.09 ± 4.81, 450.00 ± 0.01, and 52.18 ± 0.64, respectively (Figure 1).

For intracellular amastigote forms, EHA, OGSA, and SAP exhibited IC_50_ values of 181 ± 8.12, 52.11 ± 7.63, and 13.98 ± 1.43 *μ*g/mL, respectively. The cytotoxicity for the macrophages and intracellular parasites was compared using the selectivity index (SI), which was determined by the ratio of the cytotoxic concentration 50% (CC_50_) for the J774A1 macrophages and IC_50_ for amastigotes (Table 1).

If SI values are higher than 1.0, the sample tested is selective for* L. amazonensis* promastigotes, since the concentration of drug required to decrease protozoa viability is lower than that needed to destroy the mammalian cells. Among the samples of* S. saponaria* L., only the OGSA fraction was selective for the parasite.

Hemolytic activity differed among OGSA, EHA, and SAP. When tested at 100 *μ*g/mL (a value close to the OGSA IC_50_), test drugs showed 0, 22.51 ± 1.07, and 100% of hemolysis for OGSA, EHA, and SAP, respectively. At the highest concentration tested (1000 *μ*g/mL), the OGSA fraction treatment resulted in only 34.52 ± 3.99% hemolysis, while EHA and SAP treatment elicited 95.37 ± 1.12% and 100% hemolytic erythrocytes, respectively.

### 3.3. Morphological and Ultrastructural Alterations in* L. amazonensis* Caused by the OGSA Fraction

**Figure 2:**
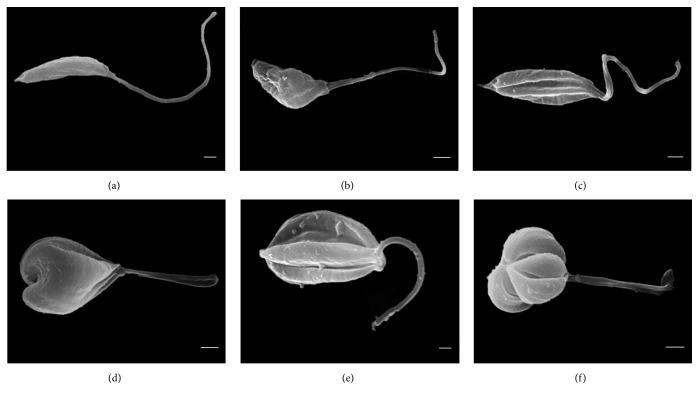
Scanning electron microscopy of promastigotes of* L. amazonensis* treated for 72 h with OGSA. (a) Untreated parasites have a typical elongated morphology. ((b) and (c)) Parasites treated with 100 *μ*g/mL of OGSA show morphological changes as cell body deformation, twisting of the flagellum, and reticent increase in volume. ((d) to (f)) Parasites treated with 450 *μ*g/mL OGSA exhibit more pronounced morphological changes. Scale bar = 1 *μ*m.

Owing to the promising results presented by OGSA, parasites treated with OGSA were subjected to electron microscopy (SEM), which revealed morphological alterations in the promastigote forms of* L. amazonensis*. Untreated microorganisms showed standard characteristics such as elongated shape, typical size, and a single flagellum. OGSA treatment modified the shape and size of the cell body, and twisted the flagellum, mainly in parasites treated at the IC_90_ (450 *μ*g/mL) of OGSA (Figure 2). Moreover, important unevenness on the cell membrane was observed in the SEM micrographs (Figures 2(b) and 2(d)), although there were no signs of cytoplasmic content extravasation.

**Figure 3:**
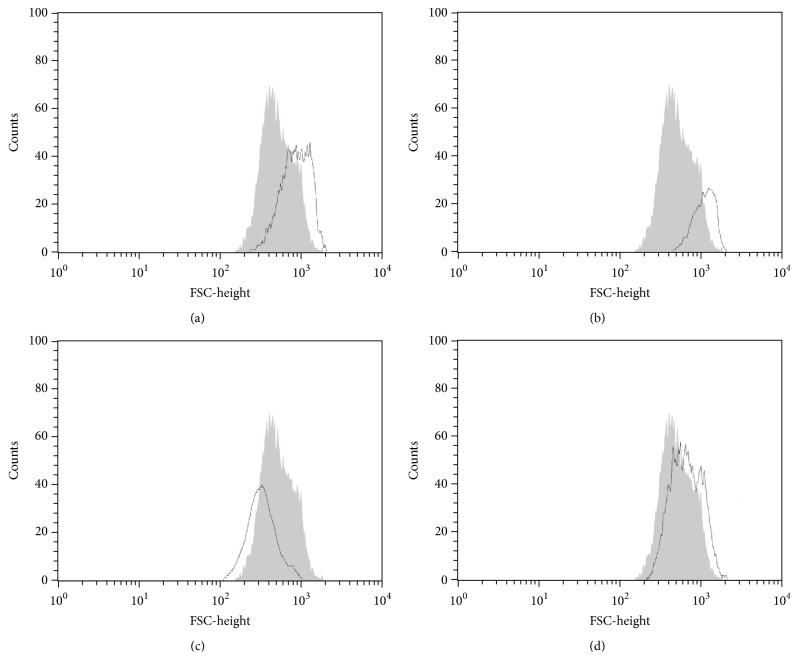
Cell volume of* L. amazonensis* promastigotes treated with OGSA fraction at concentrations of 100 *μ*g/mL (a), 450 *μ*g/mL (b), and 900 *μ*g/mL (c) and with Actinomycin D (positive control) (d). The gray area corresponds to the untreated control cells.

Flow cytometry was used to confirm the increased cell volume in promastigote forms revealed by SEM. Flow cytometry histograms showed that OGSA-treated parasites exhibited increased parasite volume (Figures 3(a) and 3(b)). Initially, a dose-dependent increase in cell volume was observed (32.15 and 76.23%, at OGSA concentrations of 100 and 450 *μ*g/mL). However, treatment with the highest OGSA concentration (900 *μ*g/mL) decreased cell volume by 28.75%, probably due to early apoptotic processes induced by the high drug concentration (Figure 3(c)).

**Figure 4:**
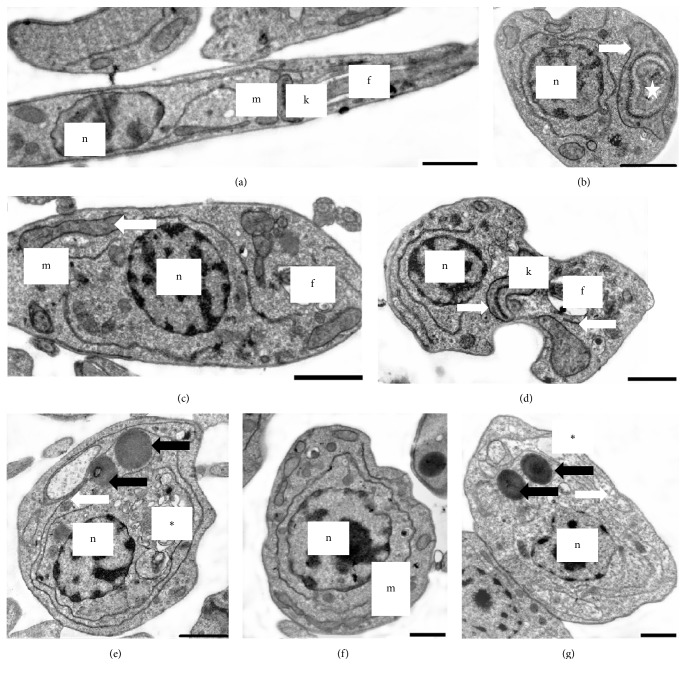
Transmission electron microscopy of* L. amazonensis* promastigotes treated for 72 h with OGSA. (a) Untreated parasites have a typical elongated morphology. ((b) to (d)) Parasites treated with 100 *μ*g/mL OGSA exhibit morphological changes. ((e) to (g)) Parasites treated with 450 *μ*g/mL OGSA exhibit more pronounced morphological changes. White arrows indicate swollen mitochondria, black arrows indicate lipid bodies, star indicates concentric structures within the mitochondria, and asterisks indicate vacuoles. f, flagellum; m, mitochondria; n, nucleus; k, kinetoplast. Scale bar = 1 *μ*m.

Transmission electron microscopy (TEM) of OGSA-treated promastigotes also indicated the presence of ultrastructural alterations (Figure 4). OGSA induced damage in parasite mitochondria, which presented disorganized features and the presence of inner concentric membrane structures. Extensive cytoplasmic vacuolization (Figure 4(e)) and general disorganization of the organelles (Figure 4(f)), mainly related to the endoplasmic reticulum (Figures 4(b), 4(e), and 4(g)), were important changes caused by OGSA treatment. Alterations in the nucleus, number of flagella, or cytoplasmic membrane were not found by TEM analysis.

### 3.4. Alteration of Mitochondrial Transmembrane Potential (ΔΨm)

**Figure 5:**
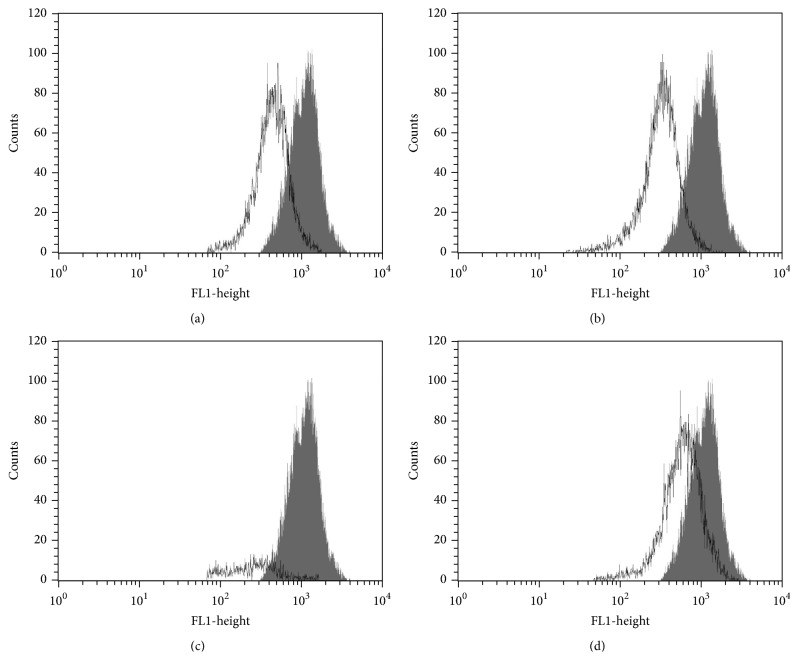
Rhodamine 123-labeled mitochondrial membrane potential assay by flow cytometry. The figure shows* L. amazonensis* promastigotes treated with 100 *μ*g/mL (a), 450 *μ*g/mL (b), and 900 *μ*g/mL (c) and with CCCP (positive control) (d). The gray area corresponds to the untreated control cells.

TEM demonstrated that OGSA treatment altered parasite mitochondria, and the ΔΨm was evaluated by flow cytometry using Rh 123, which accumulates inside healthy mitochondria. The histograms revealed a decrease in total Rh 123 fluorescence intensity upon OGSA treatment, demonstrating that Rh 123 was not inside the mitochondria, probably due to mitochondrial membrane depolarization. OGSA at concentrations of 100, 450, and 900 *μ*g/mL caused 46.23, 60.04, and 75.63% decreases in total Rh 123 fluorescence intensity, respectively, compared with the gray area, representing the untreated control cells (Figure 5).

### 3.5. Induction of Phosphatidylserine Exposure

**Figure 6:**
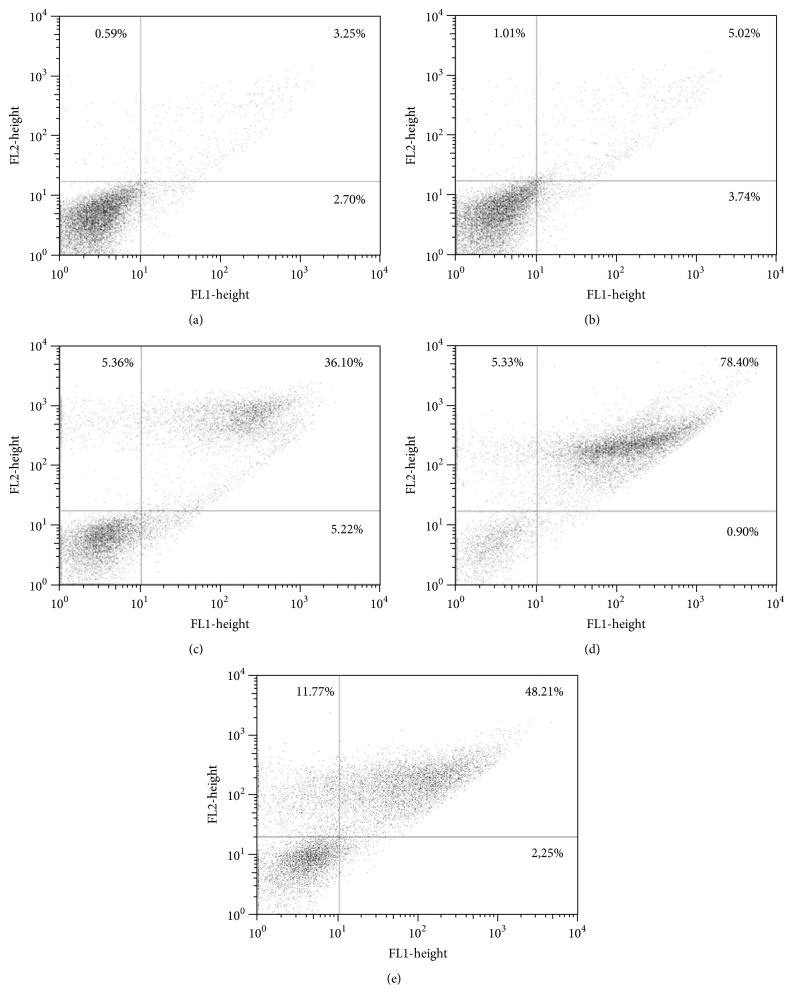
Phosphatidylserine exposure in untreated* L. amazonensis* promastigotes (a) and promastigotes treated with 100 *μ*g/mL (b), 450 *μ*g/mL (c), and 900 *μ*g/mL (d) OGSA for 24 h using annexin-V FITC and PI. Miltefosine was used as positive control (e).

Phosphatidylserine (PS) is a phospholipid that, under normal biochemical conditions, remains on the inner face of the plasma membrane or cytosolic face. During the apoptotic process, these molecules are translocated to the cell surface, working as a signal to be phagocytosed from defense cells. Annexin-V has an affinity for phosphatidylserine and the fluorescence emitted from FITC conjugated to annexin-V suggests apoptotic or late apoptotic processes if propidium iodide fluorescence is negative or positive, respectively. The typically labeled parasites appeared at the upper and lower-right quadrants on the dot-plot graphic. The intensity of annexin-V and PI fluorescence was increased up to 78.40% compared to intensity of untreated parasites, suggesting a late apoptotic process (Figure 6). Annexin-V-FITC was able to link to the phosphatidylserine on the inner face of the cell membrane, indicating the loss of membrane integrity, which can be observed by the PI fluorescence.

## 4. Discussion

Current treatments for leishmaniasis are unsatisfactory because the parasite is also a eukaryotic organism and shares many features with its mammalian cell hosts [25]. Moreover, there has been an increase in the number of cases resistant to the available drugs recommended for treating cutaneous and visceral leishmaniasis. It is imperative to search for new, effective, safe, low-cost agents to replace the current ones [26].

The use of plants as medicine involves the investigation of active compounds, starting with the isolation of morphine from opium in the 19th century [27, 28]. Methods such as fractionation, enrichment of certain substances, isolation, and characterization of pharmacologically relevant compounds from medicinal plants are currently being applied. In addition, drug research techniques have been used to standardize herbal formulations and elucidate their compounds considered as analytical markers [29].

In the present study, EHA, SAP, and OGSA exhibited antiproliferative effects on promastigotes and intracellular amastigotes, along with reduced survival of intracellular parasites in macrophages. However, OGSA was the only fraction more selective for the protozoan than for mammalian cells, and at 100 *μ*g/mL, close to its IC_50_ for promastigotes, did not show any degree of hemolysis. In contrast, the hemolytic activity of SAP was present even at low concentrations, as expected. Saponins are known to disrupt erythrocytes by interacting with sterols on erythrocyte membranes, increasing cell permeability and causing hemoglobin loss [30].

For these reasons, OGSA was selected and subjected to additional investigations to study the effects of this compound on promastigote forms of* L. amazonensis*.

OGSA acted like an antileishmanial substance by affecting the parasite's mitochondrial function even at lower concentrations, according to the variations in mitochondrial transmembrane potential (ΔΨm), shown from flow cytometry experiments using Rh 123. This potential (ΔΨm) refers to the electrochemical gradient generated by the passage of electrons through complexes I to IV of the respiratory chain, resulting in the accumulation of protons between the external and internal mitochondrial membranes. The return of these protons to the mitochondrial matrix through complex V allows the phosphorylation of ADP to ATP, the main cellular energy source [31]. Rhodamine 123 accumulates in an inverse proportion to ΔΨm, and it may be an indicator of cell damage. Maintaining transmembrane potential is essential for parasite survival [32].

Flow cytometry analysis indicated a late apoptotic process or necrosis after treatment with the higher concentration of OGSA. Based on flow cytometry alone, it cannot be determined which of these scenarios is occurring, since it is not possible to conclude if the necrotic process was primary or secondary [33].

Apoptotic events may precede cell death. Following the translocation of phosphatidylserine, apoptotic cells lose the integrity of their plasma membrane [34]. OGSA seems to dose-dependently induce this phenomenon, which was revealed by PI experiments. An overall analysis of the results suggests that the cellular effects of the lower concentrations of OGSA caused a mild necrotic process, a possible primary effect evidenced by cell swelling and positive PI results. Nevertheless, SEM and TEM analyses did not reveal intracellular content extravasation or decreased intracellular density.

The highest concentration of OGSA seemingly caused an opposite effect, indicated by cell shrinkage and other evidence of apoptosis, like cytoplasmic vacuolization and organelle disorganization, mainly in the mitochondria and endoplasmic reticulum, according to TEM micrographs and Rh 123 labeling, respectively [35]. These apoptotic features appeared gradually and dose-dependently, eventually reaching late apoptosis and cell death by loss of cell membrane integrity, causing changes in the osmotic balance between the parasites and the environment and jeopardizing the selective permeability of the plasma membrane. These interpretations allow us to suggest that necrosis was a secondary effect from the highest concentration of OGSA treatment.

In addition, evidence of autophagy, like typical autophagosomes, was not found from TEM analysis, which is considered the gold standard to study this process [36]. However, the intense disorganization of the endoplasmic reticulum might create these structures after a longer time of OGSA exposure past 24 h.

Other glycosides have been reported as cell death inducers in some studies; however, they always present some cyclization in their chemical structure. The acyclic sesquiterpene oligoglycosides studied in this paper do not possess any kind of cyclization but still induced cell death, which may show that their mechanism of action is distinct. Secoiridoid glycosides from* Swertia chirata* cause protozoa cell death by inhibiting the catalytic activity of* L. donovani* topoisomerase I [7]. A tetrasaccharide antigen found on the lipophosphoglycan membrane of the genus* Leishmania* may be explored using a synthetic carbohydrate-based vaccine for the treatment of leishmaniasis [37]. The quinovic acid glycosides isolated from* N. diderrichii* show strong activity against intracellular amastigotes of* L. infantum,* and the mechanism in this case was inhibition of parasite internalization in the promastigote form [8]. In addition, cardiac glycosides are exceptional inducers of immunogenic cell death, an effect that is associated with inhibition of the plasma membrane Na+/K+ ATPase [38].

In contrast, monodesmosidic saponins, such as those present in* S. saponaria* L. [39], act specifically on the plasma membrane of* L. infantum *promastigotes by nonspecific interactions with the ergosterol present in these parasites. This interaction causes loss of integrity or changes the negative charge of the carbohydrate portion of cell membranes. This peculiar mechanism of action of saponins, which act exclusively on the cell membrane and their integrity to cause necrotic processes, may explain the difference between SAP and OGSA regarding their antiproliferative activity, the effects on intracellular amastigotes, and toxicity in general, since the OGSA fraction greatly affected apoptotic process and the development of cell death.

When biological activities of vegetal species are investigated, it is important to determine which plant components are responsible for the observed activity. In addition, it is important to verify if the crude extract is more or less active than its fractions. Besides the investigation of the antiprotozoal and cytotoxic activities of a given sample, it is necessary to consider other factors to elucidate the effects of compounds tested on the parasites. Thus, the search for morphological, ultrastructural, and biochemical changes is important to help determine the probable mechanism responsible for cell death.

## 5. Conclusion

Our results demonstrated inhibition of promastigote and intracellular amastigote forms of* L. amazonensis *from the extract and fractions of* S. saponaria *L. However, only the OGSA fraction was selective for different evolutionary forms of* L. amazonensis *and did not show hemolytic activity. OGSA induced morphological and ultrastructural alterations in promastigote forms, and these results were confirmed by cytometry assays. Thus, our findings suggest that treating with high concentrations of the OGSA fraction can have an antileishmanial effect, inducing apoptotic processes followed by necrosis.

## Figures and Tables

**Table 1:** Cytotoxic and antiparasitic effect of *S. saponaria* L. on J774A1 macrophages and *L. amazonensis* forms, respectively.

*S. saponaria* samples	Cytotoxicity CC_50_ (*μ*g/mL)	IC_50_ intracellular amastigotes (*μ*g/mL)	SI intracellular amastigotes	IC_50_ promastigotes (*μ*g/mL)
EHA	81.66 ± 2.88	181 ± 8.12	0.45	153.70 ± 3.20
OGSA	383.33 ± 14.43	52.11 ± 7.63	7.35	100.92 ± 1.56
SAP	2.00 ± 0.7	13.98 ± 1.43	0.14	25.41 ± 2.88
